# Social Fever or General Immune Response? Revisiting an Example of Social Immunity in Honey Bees

**DOI:** 10.3390/insects11080528

**Published:** 2020-08-13

**Authors:** Michael Goblirsch, Jenny F. Warner, Brooke A. Sommerfeldt, Marla Spivak

**Affiliations:** Department of Entomology, Bee Research Facility, University of Minnesota, 1980 Folwell Avenue, St. Paul, MN 55108, USA; fors0057@umn.edu (J.F.W.); bsommerf@umn.edu (B.A.S.)

**Keywords:** chalkbrood disease, febrile response, honey bee pathogens, host-pathogen interaction, social behavior, social immunity

## Abstract

**Simple Summary:**

Behavioral, or social fever in honey bees is frequently cited as a form of social immunity—the behavioral, organizational, and physiological mechanisms that social organisms use to defend against parasites and pathogens to maintain group health. It has been shown previously that colonies elevate brood nest temperature as a response to challenge with the fungal pathogen, *Ascosphaera apis*, the causative agent of chalkbrood disease in honey bees. Our objective was to test whether we could replicate social fever and its effect on reducing signs of chalkbrood disease in colonies using methods similar to previous reports. We affirmed that honey bees increase the temperature of the brood nest after exposure to *A. apis*. However, the magnitude of temperature increase was insufficient at preventing infection, as all colonies showed signs of chalkbrood post-exposure. We conducted additional studies to explore alternative hypotheses related to the cause and effect of behavioral fever. We found that challenge with *A. apis* resulted in an increased immune response of adult bees, but this activation was not due to thermal and other stress, as measured by expression of the heat stress and nutritional genes, *Hsp 70Ab-like* and vitellogenin, respectively. We proposed additional hypotheses that could be tested.

**Abstract:**

Honey bees use several strategies to protect themselves and the colony from parasites and pathogens. In addition to individual immunity, social immunity involves the cumulative effort of some individuals to limit the spread of parasites and pathogens to uninfected nestmates. Examples of social immunity in honey bees that have received attention include hygienic behavior, or the removal of diseased brood, and the collection and deposition of antimicrobial resins (propolis) on interior nest surfaces. Advances in our understanding of another form of social immunity, social fever, are lacking. Honey bees were shown to raise the temperature of the nest in response to temperature-sensitive brood pathogen, *Ascosphaera apis*. The increase in nest temperature (−0.6 °C) is thought to limit the spread of *A. apis* infection to uninfected immatures. We established observation hives and monitored the temperature of the brood nest for 40 days. This observation period was broken into five distinct segments, corresponding to sucrose solution feedings—Pre-Feed, Feed I, Challenge, Feed II, and Post-Feed. *Ascosphaera apis* was administered to colonies as a 1% solution of ground sporulating chalkbrood mummies in 50% *v*/*v* sucrose solution, during the Challenge period. Like previous reports, we observed a modest increase in brood nest temperature during the Challenge period. However, all hives presented signs of chalkbrood disease, suggesting that elevation of the nest temperature was not sufficient to stop the spread of infection among immatures. We also began to explore the molecular mechanisms of temperature increase by exposing adult bees in cages to *A. apis*, without the presence of immatures. Compared to adult workers who were given sucrose solution only, workers exposed to *A. apis* showed increased expression of the antimicrobial peptides abaecin (*p* = 0.07) and hymenoptaecin (*p* = 0.04), but expression of the heat shock response protein *Hsp 70Ab-like* (*p* = 0.76) and the nutritional marker vitellogenin (*p* = 0.72) were unaffected. These results indicate that adult honey bee workers exposed to a brood pathogen elevate the temperature of the brood nest and initiate an immune response, but the effect of this fever on preventing disease requires further study.

## 1. Introduction

Immature honey bees require the support of their adult sisters for feeding, hygiene, and regulating the nest microclimate (e.g., temperature, relative humidity). As immatures are incapable of generating appreciable amounts of heat, they rely on the thermoregulatory behaviors of their adult caregivers to maintain a stable nest temperature during their development [1,2,3,4]. When immatures are present, adult workers maintain the temperature of the brood area within a relatively narrow range (33–36 °C) [5]. Rearing of immatures at suboptimal temperatures can lead to abnormalities that are expressed upon adult eclosion. These abnormalities manifest themselves in learning and memory [6,7,8], morphology [9,10], task specialization and performance [11], stress resistance [12], reproductive physiology [13], and longevity [12,13,14].

Besides developmental effects, immature honey bees reared at suboptimal temperatures might be more susceptible to certain pathogens [15]. For example, chalkbrood disease caused by the fungus, *Ascosphaera apis*, is a stress disease of weak or small colonies [16]; for a review, see [17]. Adult bees are not thought to be affected by *A. apis* but transmit fungal spores to larvae via contaminated brood food. Once consumed, the spores germinate in the larval gut. The subsequent vegetative growth infiltrates the hemocoel and tissues, resulting in death. What remains of the dead larva is a characteristic “mummy” that contains infectious spores [17]. The infection and growth of *A. apis* in honey bee larvae are affected by environmental conditions of the brood nest area. Below-normal brood temperature and high relative humidity can produce disease and accelerate mortality in a large percentage of larvae exposed to the pathogen [18,19]. Although the exact triggers that induce changes in behavior are unknown, adult workers might respond to the presence of *A. apis* with added brood care directed towards mitigating the negative effects of the chalkbrood disease. After experimental inoculation of small colonies with *A. apis* [20], a modest (but significant) deviation from expected brood temperature (+0.56 °C) was observed, which suggested that adult workers generate a behavioral fever. *Ascosphaera apis* is temperature-sensitive; therefore, the adult-driven increase in brood nest temperature was hypothesized to kill the pathogen and prevent its spread among uninfected immature nestmates [20,21].

Behavioral fever in honey bees, often referred to as social fever [22], is frequently cited as a form of social immunity [23,24,25,26]. Social immunity involves the behavioral, organizational, and physiological mechanisms that social organisms use to defend against parasites and pathogens to maintain the health of the group [23]. Although all colonies tested in [20] showed increased brood temperature after challenge, only one colony developed minor signs of chalkbrood disease, which was cited as evidence of an active defense against the pathogen. However, the extent to which behavioral fever limits pathogen transmission and increases colony health requires further study. We followed the experimental methods of [20], where our objective was to determine if we could replicate behavioral fever and its effect on reducing pathogen load in colonies challenged with *A. apis*. After finding that bees’ elevation of nest temperature was not sufficient to limit the establishment of chalkbrood disease, we explored some immune and stress response mechanisms that might underlie the observed temperature increase.

## 2. Materials and Methods

### 2.1. Observation Hive Experiment

#### 2.1.1. Hive Setup

We adopted an experimental design like [20], with some exceptions. Colonies were started from packages with new queens on 16 April 2018. On 31 May 2018, the queen, a frame containing mixed ages of brood, an empty frame of honeycomb, and 2500–3000 adult workers were transferred from each of the three field colonies into two-frame observation hives. A section of queen excluder material was inserted between the upper and lower frames that restricted the queen’s egg-laying to a lower brood area, yet allowed space for the workers to store incoming nectar and pollen in the empty comb cells of the upper frame. We placed the observation hives in a darkened, temperature-controlled room, which was in the Bee Research Facility at the University of Minnesota. The sides of the observation hives were lined with 5.08 cm-thick Styrofoam paneling, to provide a temperature buffer between the internal colony environment and the external surrounding room. Polypropylene tubing (id of 2.54 cm) connected the observation hives to the external environment, permitting the bees to exit and enter the hives freely.

#### 2.1.2. Temperature Recording

A temperature sensor was embedded into the wax comb at the center of the brood nest area (Brood) and the upper frame (Non-Brood, data not shown) for each colony. Each sensor was connected to an RC-4 mini temperature data logger (Elitech, Milpitas, CA, USA). The data loggers were calibrated by submerging the sensors in ice water prior to the start of the experiment, and had an accuracy of ±0.5 °C. The temperature at the two positions within the hives was recorded every 5 min throughout the duration of the experiment. A separate data logger recorded the temperature of the room where the hives were stationed (Room). The temperature of the external environment (Ambient) was retrieved from an online weather database with the St. Paul, MN Downtown-Holman Station chosen as the locality reference point (www.wunderground.com/history/daily/us/mn/st.-paul). Temperature data were collected continuously from 15:00 1 June 2018 until 15:00 9 July 2018, when the experiment was stopped. The 5-min temperature readings were aggregated every hour and used to compute the mean temperature for each hour of the experiment, which were then applied in subsequent statistical analyses. When summarized (i.e., Figures 2 and 3), the average daily temperature was computed from the corresponding mean hourly temperatures for each day. When there was a change from one observation period to the next (e.g., Pre-Feed to Feed I, see below), the hourly temperatures starting at 0:00 on the day of the change up to the moment the change occurred were treated as a distinct day, and the hourly temperatures starting immediately after and up to and including 23:00 on the day of the change were added to the first full day of the new, subsequent period.

#### 2.1.3. Experimental Periods

The experiment was divided into five distinct observation periods: (A) Pre-Feed, colonies were unfed from 15:00 June 1 until 11:00 June 10 (n = 212 hourly observations); (B) Feed I, colonies were fed 50% *v*/*v* sucrose solution in feeders placed over ventilation screens at the top of observation hives from 11:00 June 10 until 11:00 June 20 (n = 240 hourly observations); (C) Challenge, colonies were fed 1% *w*/*v* ground sporulating chalkbrood mummies in sucrose solution from 11:00 June 20 until 15:00 June 28 (n = 196 hourly observations); (D) Feed II, colonies were again fed sucrose solution from 15:00 June 28 until 15:00 July 2 (n = 96 hourly observations); (F) Post-Feed, colonies were unfed from 15:00 July 2 until 15:00 July 9 (n = 212 hourly observations). The infectivity of the *A. apis* solution was confirmed by inoculating a field colony and observing signs of chalkbrood disease prior to testing on the observation hives. The total volume of the *A. apis* inoculum given to the colonies during the Challenge period was 200 mL per colony. The Feed I and II periods served as controls for the effect of feeding *A. apis* in sucrose solution. The Feed II period was not part of [20] but was added in our study to control for a nectar dearth. At the end of the Challenge period, the colonies were graded qualitatively for severity of chalkbrood disease, using the scoring system of [27] as a guide.

### 2.2. Regression Analyses

The relationship between the hourly ambient temperature and the observed hourly temperature of the brood area for each colony was used to establish regression equations. These regression equations were then used to predict the expected temperature of the brood area for any ambient temperature, as per [20]. Since the observation hives were maintained in a temperature-controlled room, we also used the relationship between the ambient temperature and the observed hourly room temperature to determine the prediction power of using ambient temperature to establish the expected hourly brood temperature.

### 2.3. Cage Experiment

Frames containing sealed worker pupae from healthy colonies, without signs of disease were placed in an incubator at 32 °C overnight. The following day, 100 newly emerged workers were placed in 11 × 11 × 11 cm screened cages and given ad libitum access to 50% *v*/*v* sucrose solution and sterile tap water, then incubated under the same temperature conditions. Seven days after introduction into cages, the sucrose solution was exchanged with a 1% *w*/*v* solution of ground chalkbrood mummies in sucrose solution in one set of cages (Challenge; n = 3), while the sucrose solution was replaced with fresh sucrose solution in a second set of cages (Sucrose; n = 3). Bees were given ad libitum access to their respective feeds during the next 7 days.

### 2.4. Quantitative Reverse Transcription PCR (RT-qPCR)

A subsample of adult workers (n = 8/cage) was collected at 7 and 14 days after being introduced into cages when they were ≤1 day old. The timing of the collection corresponded to both the adult age of the workers and the end of each 7-day treatment period. Workers were frozen immediately in liquid nitrogen and then archived at −80 °C until RNA extraction. Total RNA was extracted from individual whole bees using TRIzol reagent, according to the manufacturer’s protocol (Invitrogen, Carlsbad, CA, USA). Quality and quantity of the extracted RNA was evaluated using a NanoPhotometer (Implen, Westlake Village, CA, USA), and cDNA was synthesized using 1 µg RNA from each worker via a two-step process. In brief, total RNA was first treated for contaminating DNA and RNases with DNase I and RNaseOUT, using a Bio-Rad T100 Thermal Cycler (Bio-Rad, Hercules, CA, USA) set to run at 37 °C for 30 min, followed by 75 °C for 10 min. In the second step, mRNA was selected and reversed transcribed using a master mix containing oligo dT_12–18_, dT_18_, dNTPs, 0.1M DTT, and SuperScript II reverse transcriptase (Invitrogen, Carlsbad, CA, USA) in 5× first strand buffer. The reverse transcription reaction was run at 42 °C for 50 min, followed by 70 °C for 15 min. Relative gene expression was quantified for the antimicrobial peptides abaecin (F 5′-CAGCATTCGCATACGTACCA-3′; R 5′-GACCAGGAAACGTTGGAAAC-3′) and hymenoptaecin (F 5′-CTCTTCTGTGCCGTTGCATA-3′; R 5′-GCGTCTCCTGTCATTCCATT-3′), the heat shock response protein *Hsp 70Ab-like* (F 5′-TATCATCGAAGCGACGACCG-3′; R 5′-TATCATCGAAGCGACGACC-3′), and the antioxidant/storage protein vitellogenin (F 5′-AGTTCCGACCGACGACG-3′; R 5′-TTCCCTCCCACGGAGTCC-3′), using previously published and validated primers. Relative expression of the target transcripts was normalized to the average of two honey bee reference genes, *β-actin* (F 5′-TTGTATGCCAACACTGTCCTT-3′; R 5′-TGGCGCGATGATCTTAATTT-3′) and ribosomal protein S5 (*RPS5*; F 5′-AATTATTTGGTCGCTGGAATTG-3′; R 5′-TAACGTCCAGCAGAATGTGGTA-3′).

### 2.5. Statistical Analysis

The difference in observed and expected temperature of the brood nest area was compared between the different observation periods among the three observation hives using a mixed model, repeated measures analysis. Observation period and time (hours) were selected as the fixed effects and colony was selected as a random effect. An autoregressive correction (AR1) was applied to the repeated structure of time, to account for possible serial correlation from consecutive sampling. Relative levels of gene transcripts of adult bees in cages were compared using a two-way ANOVA, with treatment and period as fixed effects and cage as a random effect. Data were analyzed using JMP Pro 14 (SAS Institute, Cary, NC, USA) and R 3.5.0 (R Core Team, Vienna, Austria, http://www.R-project.org/). Summary data are reported as means ±95% confidence interval.

## 3. Results

### 3.1. The Variability in Observed Brood Temperature Was Not Influenced by the Ambient Temperature

**Figure 1:**
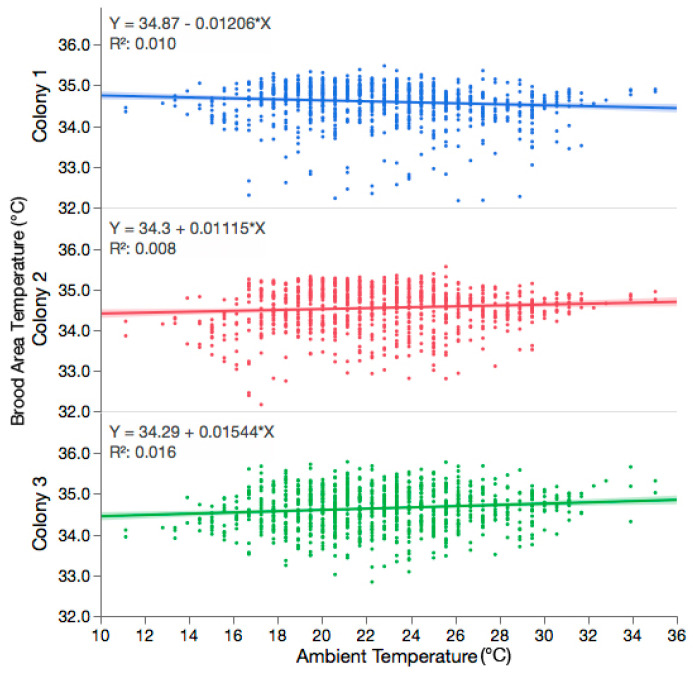
Regression analysis showing the relationship between observed hourly brood area temperature (Y) and hourly ambient temperature (X) for each colony. There was no significant correlation between observed brood temperature and ambient temperature for Colony 1 (y = 34.87 − 0.01206x, df = 954, r^2^ = 0.010, *p* = 0.0017), Colony 2 (y = 34.30 + 0.01115x, df = 954, r^2^ = 0.008, *p* = 0.0069), or Colony 3 (y = 34.29 + 0.01544x, df = 953, r^2^ = 0.016, *p* < 0.0001).

**Figure 2:**
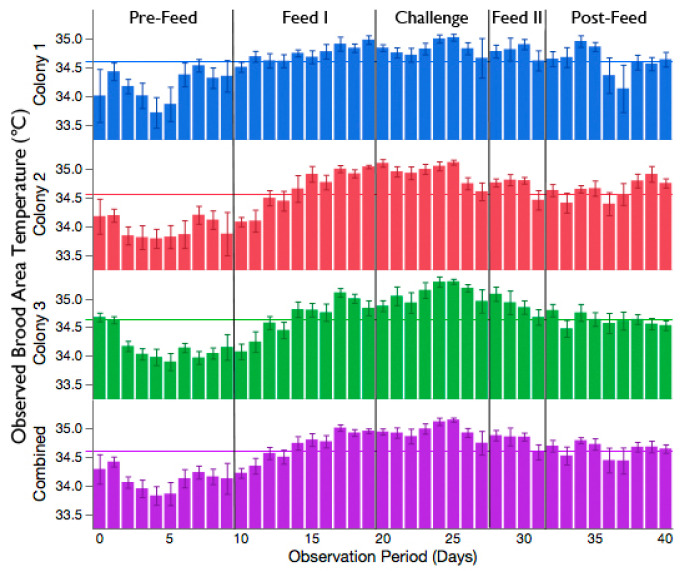
The daily observed temperature of the brood area for each colony and the average of the three colonies combined. Vertical lines mark the boundaries between the observation periods. Horizontal lines signify the overall mean observed temperature of the brood area for each colony and the three colonies combined. Each bar represents the mean daily temperature ±95% CI.

The mean observed brood temperature (±95% CI) during the experiment was 34.60 ± 0.06 °C, 34.54 ± 0.07 °C, and 34.64 ± 0.06 °C for colony 1, 2, and 3, respectively. There was no significant correlation between the observed temperature of the brood area and the ambient temperature for each colony; Figure 1 (Colony 1: y = 34.87 − 0.01206x, df = 954, r^2^ = 0.010, *p* = 0.0017; Colony 2: y = 34.30 + 0.01115x, df = 954, r^2^ = 0.008, *p* = 0.0069; Colony 3: y = 34.29 + 0.01544x, df = 953, r^2^ = 0.016, *p* < 0.0001). The observed temperature of the brood area followed a similar trend across the observation periods, even without correcting for the influence of ambient temperature; Figure 2. Despite the lack of relationship between the observed brood temperature and the ambient temperature, the regression equations were used to calculate the expected brood temperature for any given ambient temperature for each colony, as per [20]. Moreover, there was no relationship between the observed–expected brood area temperature and the ambient temperature for each colony (Colony 1: y = 0.4228 − 0.0122x, df = 954, r^2^ = 0.010, *p* = 0.0017; Colony 2: y = −0.3842 + 0.01102x, df = 954, r^2^ = 0.008, *p* = 0.0069; Colony 3: y = − 0.5248 + 0.01521x, df = 953, r^2^ = 0.016, *p* < 0.0001). The mean temperature of the room where the colonies were housed throughout the experiment was 21.79 ± 0.09 °C. Housing the colonies in this space effectively controlled for the influence of ambient temperature on the brood area.

### 3.2. Increased Brood Nest Temperature but No Reduction in Signs of Chalkbrood Disease after Challenge with A. apis

**Figure 3:**
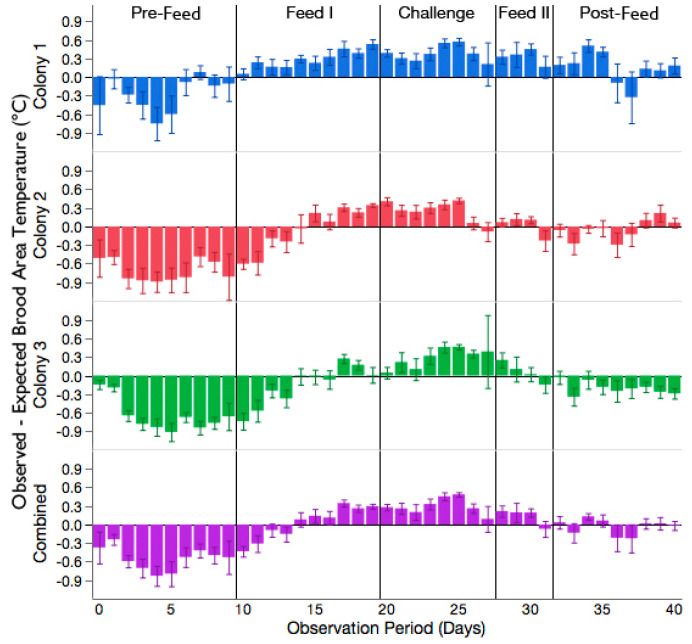
The daily difference in observed–expected temperature of the brood area for each colony and the average of the three colonies combined. A value less than 0 demonstrates that the observed temperature of the brood area was less than the expected temperature and a value greater than 0 demonstrates that the observed temperature of the brood area was greater than the expected temperature. Vertical lines mark the boundaries between the observation periods. Each bar represents the mean daily difference ±95% CI.

There was a significant interaction of the observation period and time on the difference in observed–expected brood temperature (F_4,2870_ = 15.50, *p* < 0.0001). All three colonies showed a similar trend in brood temperature change during the experiment; Figure 3. Feeding colonies sucrose solution, either before (Feed I) or after challenge with *A. apis* (Feed II), led to an increase in the temperature of the brood area, compared to periods when the colonies were not given sucrose solution (Pre- and Post-Feed). The difference in the mean observed–expected brood area temperature was +0.55 °C between the Pre-Feed and Feed I periods, and +0.19 °C between the Feed II and Post-Feed periods.

The addition of *A. apis* to sucrose solution during the Challenge period led to a further increase in the brood area temperature. The difference in the average observed–expected brood area temperature was +0.32 °C between the Feed I and Challenge periods and +0.14 °C, between the Challenge and the Feed II periods. A post-hoc Tukey’s HSD was used to compare the observed–expected brood area temperature between all observation periods. The observed–expected brood area temperature recorded during the Challenge period was significantly different than the Pre-Feed and Feed I periods (t = −5.04, *p* < 0.0001 and t = 2.76, *p* = 0.046, respectively). The observed–expected brood area temperature recorded during the Challenge period was not different than the Feed II and Post-Feed periods (t = −1.30, *p* = 0.69 and t = 1.11, *p* = 0.80, respectively). Although the change in brood temperature from expected was not different among the periods after *A. apis* was introduced into the colonies, there was a steady reduction over time in the difference between the observed–expected brood temperature once the pathogen was removed (i.e., Feed II). In other words, the observed temperature of the brood area approached the expected temperature with increasing time after exposure to the pathogen. The random factor, colony, was not significant (*p* = 0.33).

Despite the significant increase in temperature observed in the brood nest area during the Challenge period, all three colonies developed signs of chalkbrood infection. Signs of infection were observed 5 days after the colonies were first provisioned with the *A. apis* inoculum. However, the severity of chalkbrood disease was not uniform among the three colonies—Colony 2 was slightly symptomatic; Colony 3 was moderately symptomatic; and Colony 1 was highly symptomatic, as approximated by the scoring system of [27]. Colony 1, whose signs of chalkbrood disease were the most severe of the three colonies, also had the largest mean increase in brood temperature from expected during the Challenge period [+0.39 °C, 95% CI (0.35, 0.43)], compared to Colony 2 [+0.27 °C, 95% CI (0.24, 0.31)] and Colony 3 [+0.26 °C, 95% CI (0.21, 0.30)].

### 3.3. Challenge of Adult Workers with the Brood Pathogen, A. apis, Leads to an Increase in Expression of Antimicrobial Peptides

**Figure 4:**
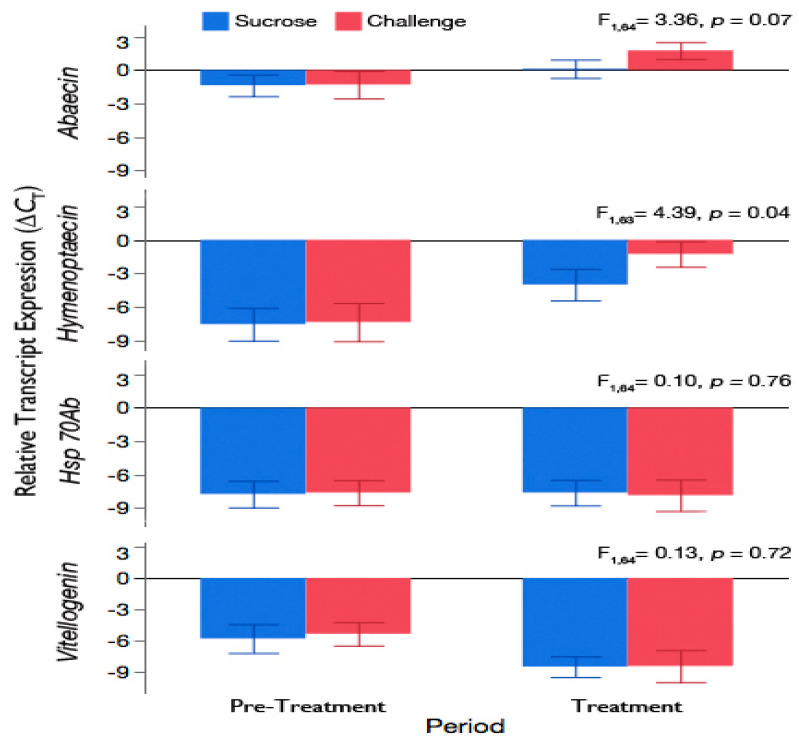
Mean transcript expression (±95% CI) of the honey bee antimicrobial peptides, abaecin and hymenoptaecin, the thermo-responsive heat shock protein 70 Ab-like (*Hsp 70Ab-like*), and the nutritional marker vitellogenin normalized to the reference genes *β-actin* and ribosomal protein S5 (*RPS5*). Newly-emerged workers were placed in cages and fed sucrose solution until day 7 (Pre-Treatment) and given either sucrose solution alone (Sucrose) or *A. apis* inoculum in sucrose solution (Challenge) from day 7–14 (Treatment). A subsample of workers was collected from replicate cages at the end of both periods (n = 7 or 8 workers/cage, 3 cages/treatment group) for the RT-qPCR analysis.

Since we observed that the increase in brood nest temperature was not sufficient to prevent infection of honey bee larvae, we next exposed workers in cages to *A. apis* to explore whether the temperature increase we saw at the colony-level could have originated through activation of underlying immune and stress physiology in adults. Expression of antimicrobial peptides were elevated in workers exposed to *A. apis* in sucrose solution compared to workers given sucrose solution alone; Figure 4. Relative levels of the fungal-specific immune gene, abaecin (F_1,64_ = 3.63, *p* = 0.07), and the bacteria-specific immune gene, hymenoptaecin (F_1,63_ = 4.39, *p* = 0.04) more than doubled for workers after 7 days of exposure to the pathogen compared to workers given sucrose solution alone. There was no effect of the random factor, cage, on abaecin (*p* = 0.43) or hymenoptaecin (*p* = 0.32) levels. Abaecin and hymenoptaecin expression were nearly identical for workers during the Pre-Treatment period of the experiment, where both groups had ad libitum access to sucrose solution (t_(34)_ = −0.08 and −0.14, *p* = 1.00, respectively).

### 3.4. Exposure of Adult Workers to A. apis Was Not Sufficient to Induce Hsp 70Ab-Like or Accelerate Downregulation of Vitellogenin Expression

Relative expression of the heat stress inducible *Hsp 70Ab-like* gene in adult workers was unaffected by exposure to *A. apis* for 7 days; Figure 4. There was no difference in expression of *Hsp 70Ab-like* between workers that were fed sucrose solution for the duration of the experiment (Sucrose) and workers whose feed was switched at day 7 to sucrose solution containing 1% ground sporulating chalkbrood mummies (Challenge; F_1,64_ = 0.10, *p* = 0.76). There was no effect of the random factor, cage, on *Hsp 70Ab-like* expression (*p* = 0.87).

Expression of vitellogenin, a marker of the nutritional state of adult honey bees was compared in workers exposed to *A. apis* in sucrose solution for 7 days (Challenge) and workers given sucrose solution throughout the 14-day duration of the experiment (Sucrose). There was no difference in the relative levels of vitellogenin between workers exposed to *A. apis* and workers given sucrose solution alone (F_1,64_ = 0.13, *p* = 0.72). Regardless of treatment, there was a decrease in the relative levels of vitellogenin over time (F_1,64_ = 24.86, *p* < 0.0001), which is expected as adult workers age [28]. There was no effect of the random factor, cage, on vitellogenin expression (*p* = 0.40).

## 4. Discussion

Starks et al. [20] laid the foundation for understanding the phenomenon of behavioral, or social fever, as a response to challenge with the fungal pathogen, *A. apis*, in honey bees. Few studies have since followed this report, describing a novel form of social immunity [21,29]. Results from our study affirmed that honey bees increase the temperature of the brood nest after exposure to *A. apis*. However, we found that the magnitude of nest temperature increase was insufficient at preventing infection, as all experimental colonies showed signs of chalkbrood infection post-exposure. As the increase in temperature of the brood nest did not prevent the spread of infection among the immatures, it is worth exploring other explanations that might account for the fever response based on our knowledge of the host–pathogen relationship at the level of both the individual and the colony.

One explanation for why we observed an increase in brood nest temperature after colonies were challenged with *A. apis* might be due to the direct activation of integrated physiological processes in adult bees. Exposure of adult workers to the pathogen might have triggered febrile signaling that lead to simultaneous stimulation of stress and immune responses [30]. Initiation of a social fever directed towards *A. apis* assumes that adult nestmates are responding to cues from infected immatures or have direct contact with the pathogen through feeding and grooming. Honey bees rely heavily on chemical cues for decision-making and behavioral adjustments. Detection of changes in a colony’s chemical profile is shown to increase worker metabolism and thermal preference towards warmer temperatures [31]. Although it is the larvae that are symptomatic for chalkbrood disease, we considered that pathogen recognition by adults, in the absence of chemical cues from infected immatures, could elicit upregulation of the adult immune system. We observed increased expression of the antimicrobial peptides, abaecin and hymenoptaecin, when *A. apis* was provisioned to adult workers in cages. We chose to measure abaecin expression, as this gene codes for a fungal-specific antimicrobial peptide [32]. We also chose to quantify the relative expression of the bacterial-responsive antimicrobial peptide, hymenoptaecin [32], to determine if any change in immune response was general or specific to fungal exposure. Increased expression of these immune genes suggests that adult workers are responsive to *A. apis*.

We based our tests of adult worker immune activation on the fact that the immune system engages in “cross-talk” with stress-responsive mechanisms in insects [33,34]. Pathogen recognition of *A. apis* in adult workers might have activated eicosanoid signaling, leading to behavioral fever at the individual level [35,36]. Eicosanoids are conserved signaling molecules that modulate physiological processes, such as inflammation, fever, and immune responses [37]. Our attempt to link immune system activation to thermal and other stress mechanisms by measuring the expression of the heat stress protein, *Hsp 70Ab-like*, and nutritional marker, vitellogenin, did not prove successful. However, the interaction of heat stress and immune gene expression in honey bees is likely contextual, being regulated under different conditions. In some cases, this interaction might be antagonistic, as was observed in the wounding response of adult workers [38]. In other instances, heat stress and immune gene expression are positively correlated, as was demonstrated in adult workers experimentally-infected with virus [39]. Given the amount of pathogen that was delivered to the small colonies, it is likely that a high number of adult workers were exposed and points to the need for further study of the linkage between the immune system and heat and other stress responses in both immature and adult honey bees.

Because larvae in our experimental colonies showed signs of chalkbrood disease after challenge, we considered additional hypotheses that might explain the increase in nest temperature. One alternative hypothesis posed by [40] is that behavioral fever might be a manipulation by the pathogen to benefit its transmission rather than an active host defense mechanism. Campbell et al. [40] explored behavioral fever as an individual immune response in adult honey bee workers infected with the fungal pathogen *Nosema ceranae*. Workers preferred warmer temperatures within the brood nest but did not have warmer thoraces when infected [40]. *Nosema ceranae* proliferation is enhanced at above-normal host temperature [41]; therefore, fever in this scenario was hypothesized to not be an active process because infected workers that showed a preference for warmer temperatures might have had high pathogen loads and likely did not reduce the risk of infection among nestmates. Interestingly, *A. apis* cultured in vitro shows comparable vegetative growth at a relatively wide range of temperatures [19]. The outcome of workers raising the nest temperature might not have direct negative effects on the actively growing stage of the pathogen but might induce changes to the physiology of uninfected immatures and potentially reduce their susceptibility to *A. apis*. This hypothesis remains to be tested.

Another hypothesis for why we observed an increase in brood nest temperature after exposure to *A. apis* could be due to the release of metabolic heat from workers responding to a nearby, abundant source of concentrated sucrose [42,43]. The significant increase in brood nest temperature during the Feed I and II periods, highlight that the metabolism of adult workers becomes elevated when they receive a low-cost reward, such as concentrated sucrose from an artificial feeder placed close to the nest [44,45]. Hypermetabolism would result in greater thermal emissions that might have artificially inflated the change from the expected brood nest temperature. The method that we and [20] used to deliver *A. apis* consisted of suspending ground chalkbrood mummies in sucrose solution and then supplying the inoculum in a feeder placed directly above the colony. Although we observed a significant difference in brood nest temperature between the Feed I and Challenge periods, the trend for above-normal temperature was sustained through all periods where sucrose was readily available (Feed I and II and Challenge). However, access to sucrose does not fully explain the observed shift in temperature. The purpose of the sucrose-only feedings was to control for the effect of administering *A. apis* in sucrose solution. We are confident that our experimental design captures a real effect from exposing colonies to *A. apis* and repeats the findings of [20]. However, since there were no parallel colonies that were given sucrose only, then perhaps the trend in temperature change was due to other factors, such as colony phenology. For example, changes in the ratio of open brood (larvae) to sealed brood (pupae) during the experiment might have affected the thermoregulatory effort put forth by the workers [46]. Further research should examine the effects of colony phenology on thermoregulation behavior, with and without the influence of *A. apis*. Additionally, direct observation of adults in colonies would help resolve how pathogen challenge might induce changes in metabolic activity or behavior of the workers to ultimately affect brood nest temperature.

## 5. Conclusions

We confirm the findings of a previous study that showed that honey bees increase the temperature of the brood nest area when exposed to the fungal pathogen, *A. apis* [20]. However, the increase in temperature that we observed was not sufficient to prevent infection, as all experimental colonies showed signs of chalkbrood disease after challenge. We conducted additional studies to explore alternative hypotheses related to the cause and effect of behavioral fever to verify if it is an active mechanism of social immunity. We found that challenge with *A. apis* resulted in an increased immune response of adult bees, but did not find that the immune activation was due to thermal and other stress, as measured by expression of the heat stress and nutritional genes, *Hsp 70Ab-like* and vitellogenin, respectively. We proposed additional hypotheses that could be tested—that the increase in nest temperature could be a manipulation by the pathogen to increase transmission, or a metabolic or physiological response of adult bees to sucrose solution feeding.
